# Ginsenoside Rg3 Adjunctively Increases the Efficacy of Gefitinib Against NSCLC by Regulating EGFR Copy Number

**DOI:** 10.3390/ph18071077

**Published:** 2025-07-21

**Authors:** Xinyi Lv, Yuehan Song, Tianhua Liu, Dingdan Zhang, Xinpeng Ye, Qingqing Wang, Rongrong Li, Jiayi Chen, Shujing Zhang, Xue Yu, Chunying Hou

**Affiliations:** School of Traditional Chinese Medicine, Beijing University of Chinese Medicine, Beijing 102488, China; 20220931063@bucm.edu.cn (X.L.);

**Keywords:** ginsenoside Rg3, EGFR, copy number, NSCLC, gefitinib

## Abstract

**Background**: Lung cancer has the highest morbidity and mortality of all tumors, and the development of TKI drugs targeting EGFR activating mutations has brought lung cancer treatment into the targeted era. In view of their low efficacy and susceptibility to drug resistance, there is an urgent need to find strategies to increase their efficacy and reduce the incidence of drug resistance. **Methods**: In this study, we examined the distribution and probability of EGFR mutations in non-small cell lung cancer patients in the cBioPortal database and compared the survival prognosis of patients with normal and abnormal EGFR, NSCLC patients treated with and without TKI, and NSCLC patients with different EGFR gene copy numbers. We established a mouse lung cancer model and examined the histomorphological characteristics of lung tissues via hematoxylin and eosin staining. Additionally, changes in the copy number of the EGFR gene and its protein expression levels were detected using RT-qPCR and Western blotting. Furthermore, we quantified the concentration of the EGFR protein using ELISA. **Results**: We found no significant advantage of EGFR-TKI therapy over first-line chemotherapeutic agents in patients with EGFR-abnormal NSCLC. The reason for this may be related to the abnormal EGFR gene copy number; the higher the copy number increases, the worse the survival prognosis of the patients. In molecular biology experiments, we demonstrated that ginsenoside Rg3 down-regulated the copy number of 18, 19, 20, and 21 exons and protein expression of EGFR in lung adenocarcinoma cells. The results of in vivo pharmacodynamic assays confirmed that sequential administration of ginsenoside Rg3 with TKI drugs could achieve a gainful complementary effect. **Conclusions**: Ginsenoside Rg3 down-regulates the copy number of EGFR important exons in EGFR-mutant cells of lung adenocarcinoma and reduces EGFR protein expression, thus providing a high gainful complementary effect in combination with EGFR-TKI.

## 1. Introduction

Lung cancer is the most prevalent type of malignancy in the world and has the highest lethality rate of all tumor types [1]. In the early stage, the treatment of lung cancer is mainly based on radiotherapy and chemotherapeutic drugs, as well as surgical interventions [2]. The development of TKI (tyrosine kinase inhibitors) drugs targeting EGFR (epidermal growth factor receptor)-activating mutations has brought lung cancer treatment into the targeted era [3].

EGFR is a receptor that binds EGF (epithelial growth factor) to promote cell proliferation and signaling, with significantly increased expression or aberrant activation in numerous solid tumors [4,5]. EGFR is a glycoprotein located on the surface of the cell membrane and belongs to the tyrosine kinase-type receptors. It was activated by binding to ligands which include EGF and TGFα (transforming growth factor α) or other members of the ErbB receptor family, such as ErbB2/Her2/neu polymerization [6,7,8]. Upon activation, EGFR converts from a monomer to a dimer and activates its kinase pathways located in the cell, including activation sites such as Y992, Y1045, Y1068, Y1148, and Y1173 [4,9]. This autophosphorylation can direct downstream phosphorylation, including MAPK [10], Akt [11], and JNK pathways, which induce cell proliferation [7]. Experimental studies have demonstrated that inhibiting the expression levels of EGFR and blocking its phosphorylation can suppress the release of exosomes from hypoxic tumor cells, thereby exerting significant therapeutic effects in the treatment of cancer [12].

Mutant EGFR is present in many tumors, and many EGFR-mutant phenotypes have been identified [13,14]. Mutants usually arise due to deletion, mutation, and rearrangement of the EGFR gene resulting in the absence of certain structural domains [15,16]. Abnormalities in specific structural domains lead to disruption of receptor downregulation mechanisms, activation of abnormal signaling pathways and inhibition of apoptosis, resulting in sustained activation of cells with ligand-independent receptors [17]. When a gene mutation occurs at a specific EGFR locus, it can cause a significant increase in the level of EGFR autophosphorylation. Increasing expression of mutant EGFR receptors or ligands leads to sustained activation of EGFR. At the same time, the high expression of EGFR causes enhanced downstream signaling, while enhanced action of the autocrine loop, disruption of receptor downregulation mechanisms and activation of aberrant signaling pathways may account for the presence of high or aberrant EGFR expression in many solid tumors [18].

The mechanism of action of EGFR-TKI drugs is to bind to EGFR tyrosine kinase activation region sites, thus blocking the downstream protein kinase B (PKB/AKT), STAT pathway, and MAPK (mitogen-activated kinase protein) activation pathway, and ultimately blocking the EGFR signaling pathway involved in growth and metastasis of tumors [3]. First-generation reversible inhibitors of EGFR tyrosine kinases, belonging to the anilinoquinazoline class, competitively inhibit the binding of ATP to the EGFR tyrosine kinase activation region site [8]. Most lung cancer patients treated with first-generation EGFR-TKI develop resistance about 1 year after treatment. T790M mutations appear in tissue specimens from approximately 49% to 63% of patients with acquired resistance and are thought to be the most important cause of resistance to first-generation TKI [5,19]. The second-generation EGFR-TKI differs from the first-generation. It binds to the tyrosine kinase activation region irreversibly, and it is also a pan-HER inhibitor. The second-generation EGFR-TKI can simultaneously inhibit EGFR, HER-2, and HER-4 receptor phosphorylation and their subsequent kinase activity [20]. Approved for metastatic NSCLC with sensitive EGFR mutations, second-generation TKIs serve as alternatives to first-generation inhibitors, though their multitarget mechanism confers stronger adverse effects that may compromise patient tolerance [8]. Third-generation TKI mainly inhibits signaling pathways by forming covalent bonds with the tyrosine kinase binding domain Cys797 and avoiding T790M [7,16]. Third-generation TKIs overcome drug resistance caused by T790M mutations and have been well tolerated [21,22,23].

Various authoritative guidelines recommend TKI treatment covering first-, second-, and third-line treatment and even maintenance therapy for advanced NSCLC (non-small cell lung cancer) [24]. For patients with advanced NSCLC, EGFR-TKI has become the standard of care for patients with progressive EGFR mutation-positive disease. Whether TKI-targeted therapeutic agents against EGFR have more significant advantages over clinically recommended first-line chemotherapeutic agents in terms of prolonging the survival of NSCLC patients remains to be validated [23]. Also, the variability of EGFR treatment effects in patients with EGFR-wild-type and EGFR-mutant NSCLC needs to be further clarified. To clarify the above issues, this study analyzed the situation of NSCLC patients in 31 studies in the cBioPortal database, and the survival of NSCLC patients with normal or abnormal EGFR, treated with TKI or not treated with TKI, and NSCLC patients with different EGFR gene copy numbers were compared for prognostic analysis based on the full exploration of the distribution and probability. We also focused on the impact of EGFR gene copy number on the survival prognosis of NSCLC patients and TKI-like drugs by in-depth analysis of EGFR abnormalities (including mutations, structural variants, and copy number alterations in NSCLC). It was also fully validated by systematic evaluation and basic research.

Among the 150 distinct ginsenoside types isolated from Panax ginseng, Rg3 stands out as the most intensively studied component in cancer research. It has also been demonstrated to exhibit therapeutic effects against various pulmonary diseases. Its potential targets are continuously being explored, and in this experiment, we identified one plausible direction through which Rg3 may exert its effects [25]. A complete evaluation of the regulatory effects of ginsenoside Rg3 on the copy number of important EGFR exons and EGFR protein expression in lung adenocarcinoma cells from the perspectives of clinical efficacy, in vivo pharmacodynamic, and molecular biology was performed. The gaining effect of ginsenoside Rg3 on EGFR-TKI combination in anti-lung-cancer therapy was fully demonstrated, and a sequential dosing regimen that was more effective than the concurrent dosing regimen was proposed.

## 2. Results

### 2.1. The Current Clinical Status of NSCLC

**Figure 1:**
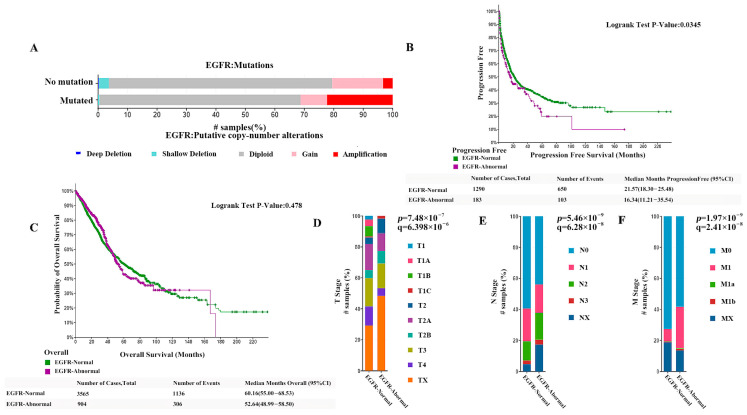
(**A**) The proportion of patients with copy number in patients with EGFR mutations. (**B**) Comparison of the progression-free survival (PFS) between patients with normal EGFR and those with abnormal EGFR. (**C**) Comparison of the overall survival rates between patients with normal EGFR and those with abnormal EGFR. (**D**–**F**) Comparison of the proportion of patients w−ith EGFR-abnormal type and the EGFR-normal type at TI, N0, and M0 stage (both *p* < 0.0001).

Among all non-small cell lung cancers, the number of samples/cases with normal EGFR was 6006/5997, the number of samples/cases with abnormal EGFR was 1570/1425, and 83 cases had crossover and were not counted in the analysis. Molecular profiles include mutations, structural variants (SV), and copy number alterations (abbreviated CNV). Among them, the number of samples/cases with EGFR mutations was 1378 (18%)/1222 (17%), the number of samples/cases with significant EGFR copy number abnormalities (amplified + homo-deleted) was 484 (6%)/461 (6%), and the number of samples/cases with structural variants was 11 (<1%)/11 (<1%). The proportion of patients with copy number amplified was significantly increased in patients with EGFR mutations, as shown in Figure 1A. In our prognostic analysis of data from patients with normal and abnormal EGFR, progression-free survival was significantly better in patients with normal EGFR than in patients with abnormal EGFR. The median progression-free survival (95% CI) of 21.57 (18.30–25.48) months in the normal EGFR group was much higher than the median progression-free survival of 16.34 (11.21–35.54) months in patients with abnormal EGFR (*p* < 0.05), as Figure 1B shows. However, the analysis of overall survival showed no significant difference between the two groups, as shown in Figure 1C. The analysis of tumor invasion and metastasis in both groups showed that the proportion of patients with the EGFR-abnormal type had a significantly lower percentage in TI stage, N0 stage, and M0 stage than those with the EGFR-normal type, as shown in Figure 1D–F. This indicates the current clinical status of NSCLC is thatcompared with patients with normal EGFR, those with EGFR abnormalities exhibit more rapid disease progression, a greater likelihood of tumor cell invasion, and significantly more severe metastasis.

### 2.2. Clinical EGFR-TKI Therapy Does Not Have a Significant Advantage over First-Line Chemotherapeutic Agents

**Figure 2:**
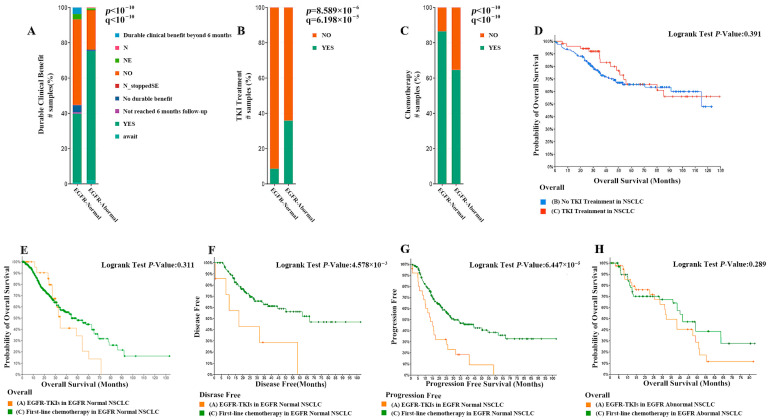
(**A**) Comparison of the proportion of patients with EGFR abnormalities and patients with normal EGFR with sustained clinical benefit (73.06% vs. 39.04%). (**B**) A total of 64.64% of patients with EGFR abnormalities underwent chemotherapy and 35.9% underwent TKI therapy. (**C**) A total of 86.44% of patients with normal EGFR underwent chemotherapy and 8.6% underwent TKI therapy. (**D**) The survival curves of NSCLC patients treated with TKI or not. (**E**) The survival curves of NSCLC patients with normal EGFR treated with TKI or not. (**F**,**G**) Comparison of disease-free survival and progression-free survival in patients treated with first-line chemotherapy versus EGFR-TKI therapy. (**H**) Overall survival curves comparing EGFR-TKI-treated patients with first-line chemotherapy in NSCLC patients with EGFR abnormalities.

A comparative analysis of the two treatment strategies showed that patients with EGFR abnormalities had a higher proportion of sustained clinical benefit compared with patients with normal EGFR (73.06% vs. 39.04%), as shown in Figure 2A. About 64.64% of patients with EGFR abnormalities underwent chemotherapy and 35.9% underwent TKI therapy (Figure 2B, *p* < 0.01). While 86.44% of patients with normal EGFR underwent chemotherapy and 8.6% underwent TKI therapy (Figure 2C, *p* < 0.01). There were significant differences in the choice of chemotherapy and TKI treatment between the two groups of patients; patients with abnormal EGFR preferred TKI treatment in their treatment strategy. To investigate whether TKI therapy has better results for NSCLC patients, we compared the survival of NSCLC patients who received TKI therapy and NSCLC patients who did not receive TKI therapy. As shown in Figure 2D, the survival curves of NSCLC patients treated with TKI were slightly higher than those of patients not treated with TKI until 52 months, but after 52 months, the two curves were essentially the same and were not significantly different from those of NSCLC patients not treated with TKI in an overall sense. Subsequently, we evaluated whether EGFR-TKI treatment had a significant advantage over first-line chemotherapy treatment in NSCLC patients with abnormal and normal EGFR genes. The results showed that among NSCLC patients with normal EGFR, the overall survival curves of patients treated with EGFR-TKI were not significantly different from those treated with first-line chemotherapy, but the former had slightly higher survival up to about 30 months than patients not treated with EGFR-TKI, and lower survival after 30 months than the latter (Figure 2E). Moreover, the disease-free survival and progression-free survival of patients treated with EGFR-TKI were significantly lower than those treated with first-line chemotherapy (Figure 2F,G, *p* < 0.01). This suggests that the efficacy of EGFR-TKI in NSCLC patients with unchanged EGFR genes is significantly lower than that of first-line chemotherapeutic agents. Among NSCLC patients with abnormal EGFR, the overall survival curves of patients treated with EGFR-TKI did not differ significantly from those treated with first-line chemotherapy, and even their tendency to have higher survival rates in the short term disappeared (Figure 2H). The above experimental results show that although NSCLC patients with abnormal EGFR prefer TKI therapy compared to those with normal EGFR, clinical EGFR-TKI therapy does not have a significant advantage over first-line chemotherapeutic agents in NSCLC patients with abnormal EGFR based on the available data.

### 2.3. EGFR Abnormalities (Including Mutations, Structural Variants, and Copy Number Alterations) in NSCLC

**Figure 3:**
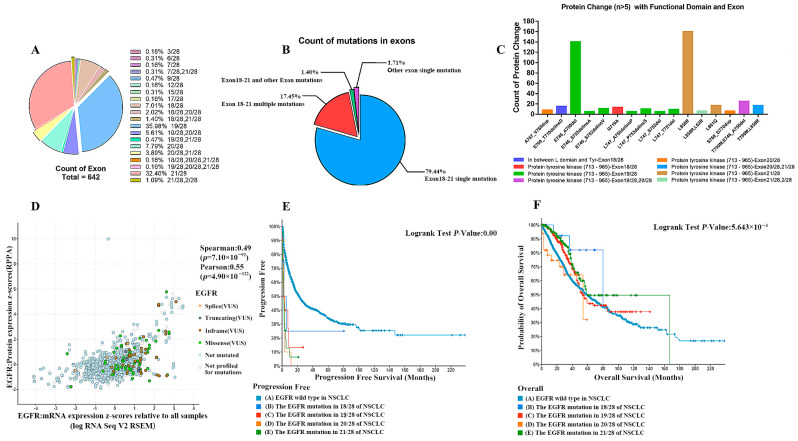
(**A**) Major distribution of mutations in EGFR exons in non-small cell lung cancer patients. (**B**) Gene mutation statistics. (**C**) The mutations of EGFR in exons 18, 19, 20, and 21 were all in the structural domain of tyrosine kinase. (**D**) Protein expression. (**E**) NSCLC patients with exon 18, 19, 20, and 21 mutations have significantly lower progression-free survival than EGFR-wild-type patients. (**F**) NSCLC patients with exon 18, 19, 20, and 21 mutations have significantly higher overall survival than wild-type patients at 50 months post-survival.

To investigate the underlying causes of the above phenomena, we further parsed EGFR abnormalities (including mutations, structural variants, and copy number alterations) in NSCLC. Classified by the site of mutation, EGFR has about 107 different mutation combinations in NSCLC. Further classification analysis of these data showed that the mutations in EGFR exons in non-small cell lung cancer patients were mainly located in 19/28 (35.98%), 21/28 (32.40%), 20/28 (7.79%), 18/28 (7.01%), and mixed mutations between the four (Figure 3A). The vast majority (79.44%) of EGFR mutations were single mutations in exons 18–21, followed by multi-locus mutations (17.45%) between exons 18–21. Very few of them were located outside of exons 18–21, whether they were single-site mutations or multi-locus multiplexes (Figure 3B). The mutations of EGFR in exons 18, 19, 20, and 21 were all in the structural domain of tyrosine kinase, and most of them were functionally acquired mutations (Figure 3C). The mutated EGFR showed a trend of slightly increased transcript levels but did not show significant trends or differences in protein expression compared with the wild type (Figure 3D). The results show that EGFR gene mutations result mainly in an increase in receptor tyrosine structural domain activity and no increase in expression. Survival analysis of NSCLC patients with exon 18, 19, 20, and 21 mutations showed that the progression-free survival of NSCLC patients with exon 18, 19, 20, and 21 mutations was significantly lower than that of patients with wild-type EGFR (Figure 3E), but the overall survival of NSCLC patients with exon 18, 19, 20, and 21 mutations was, within 50 months of survival, significantly higher than the wild-type (Figure 3F).

### 2.4. A Higher Degree of Increase in EGFR Gene Copy Number Leads to a Worse Survival Prognosis of Patients

**Figure 4:**
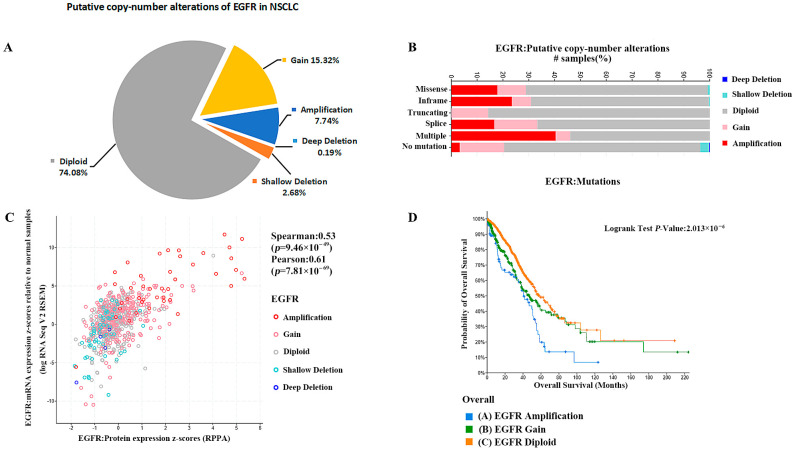
(**A**) The percentage of EGFR gene copy number deletion was 2.87%. (**B**) Analysis of EGFR mutations shows that EGFR gene copy number amplification was more likely to occur in EGFR mutant NSCLC. (**C**) The increase in gene copy number was positively correlated with the expression of EGFR. (**D**) Median months overall (95% CI) was 58.27 (52.64–73.16) for EGFR diploid patients, 46.81 (37.96–64.97) for EGFR gain patients, and 40.37 (35.10–51.30) for EGFR amplification patients.

Further analysis of EGFR gene amplification copy data from patients with non-small cell lung cancer showed that about 26% of the 3734 tested samples were detected with EGFR gene copy number alteration. The percentage of EGFR gene copy number increase was 23.06%, and the percentage of gain and amplification was 15.32% and 7.74%, respectively. The percentage of EGFR gene copy number deletion was 2.87% and the percentage of shallow deletion and deep deletion were 2.68% and 0.19%, respectively (Figure 4A). Therefore, the copy number alteration of EGFR gene in NSCLC patients was mainly increased. Furthermore, EGFR gene copy number amplification was more likely to occur in EGFR mutant NSCLC, as shown in Figure 4B. The increase in gene copy number was positively correlated with the expression of EGFR, and the expression of EGFR, including transcript level and translation level, was significantly higher in EGFR gene copy number amplification samples relative to normal diploids (Figure 4C). Prognostic analysis of data from EGFR gene copy number-altered and normal diploid patients, and overall survival analysis showed significant differences between the probability of overall survival in NSCLC patients with different EGFR gene copy numbers. As shown in Figure 4D, median months overall (95%CI) was 58.27 (52.64–73.16) for EGFR diploid patients, 46.81 (37.96–64.97) for EGFR gain patients and 40.37 (35.10–51.30) for EGFR amplification patients, which indicates that the higher degree of increase in EGFR gene copy number leads to a worse survival prognosis of patients.

### 2.5. EGFR-TKI Agents Target May Not Be Effective in Patients with a Significant Increase in EGFR Copy Number

**Figure 5:**
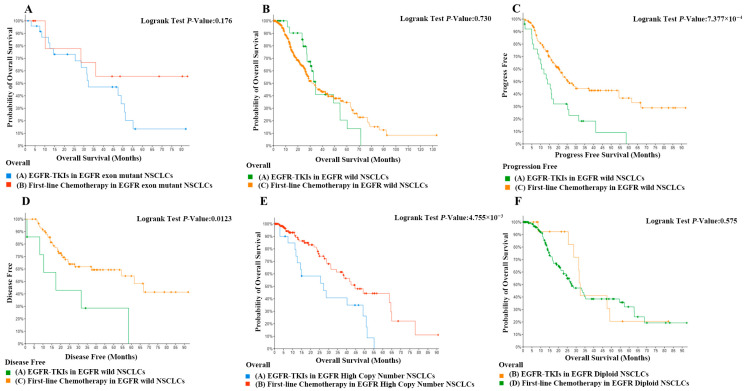
(**A**) Comparison of overall survival in first-line chemotherapy in EGFR-exon-mutant NSCLCs with EGFR-TKIs in EGFR-exon-mutant NSCLCs. (**B**) Comparison of overall survival in first-line chemotherapy in EGFR-exon-mutant NSCLCs with EGFR-TKIs in EGFR-wild NSCLCs. (**C**) Comparison of progression-free survival in first-line chemotherapy in EGFR-wild NSCLCs with EGFR-TKIs in EGFR-wild NSCLCs. *p* < 0.01. (**D**) Comparison of disease-free survival in first-line chemotherapy in EGFR-exon-mutant NSCLCs with EGFR-TKIs in EGFR-wild NSCLCs. *p* < 0.05. (**E**) Comparison of overall survival in first-line chemotherapy in EGFR-high-copy-number NSCLCs with EGFR-TKIs in EGFR-copy-number NSCLCs. *p* < 0.05. (**F**) Comparison of overall survival in first-line chemotherapy in EGFR-diploid NSCLCs with EGFR-TKIs in EGFR-diploid NSCLCs.

In this study, we utilized a matching methodology to stratify patients with stages II and III non-small cell lung carcinoma into cohorts receiving EGFR-TKIs and those not receiving TKIs. The age spectrum of the enrolled subjects extended from 37 to 82 years, with no statistically significant differences in age distribution observed between the two cohorts. Additionally, the gender composition was balanced across groups, ensuring the comparability of the study. The study meticulously selected patients with pulmonary adenocarcinoma and systematically excluded confounding variables that could potentially bias the interpretation of results, including age, gender, pathological classification, and disease staging. Through these rigorous matching and exclusion criteria, the aim was to establish a homogeneous study population to more precisely evaluate the therapeutic efficacy of TKIs within a specific subset of NSCLC patients. Then, we discussed the difference in efficacy of EGFR-TKI in NSCLC patients with EGFR mutations and increased EGFR copy numbers, respectively. The comparison of overall survival rates between patients with EGFR mutations treated with EGFR-TKI therapy and those treated with first-line chemotherapy showed no significant difference (Figure 5A). The analysis of overall survival in patients with EGFR-wild-type tumors undergoing treatment with EGFR-TKI versus first-line chemotherapy demonstrated no statistically significant differences (Figure 5B). However, the progression-free survival and probability of overall survival of EGFR-wild-type patients treated with EGFR-TKI were significantly lower than those of EGFR-wild-type patients (Figure 5C,D, *p* < 0.05). In patients with high EGFR copy number, the probability of overall survival rate was significantly lower in patients treated with EGFR-TKI than in patients receiving first-line chemotherapeutic agents (Figure 5E, *p* < 0.05). In contrast, among patients with normal EGFR copy number, there was no significant difference in probability of overall survival between patients treated with EGFR-TKI and first-line chemotherapy (Figure 5F). These results suggest that an increase in EGFR copy number is prognostically harmful in NSCLC patients, but EGFR-TKI agents target mainly tyrosine kinase receptor activity and may not be effective in patients with a significant increase in EGFR copy number. When treating patients with EGFR mutations using EGFR-TKI agents, it is essential to take into account the impact of copy number alterations and incorporate drugs that can modulate these changes, given the significant overlap between EGFR mutations and high copy numbers.

### 2.6. Ginsenoside Rg3 May Be More Sensitive for Patients with an EGFR Mutant Phenotype and EGFR May Be an Important Target for Its Action

**Figure 6:**
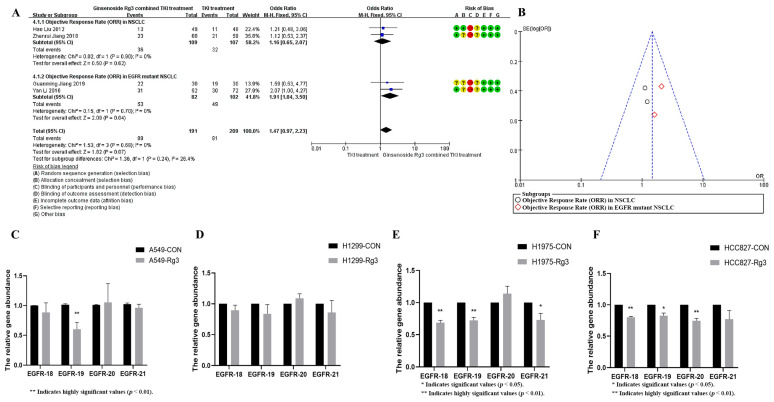
(**A**) Ginsenoside Rg3 could increase the objective remission rate of TKI-treated patients up to 1.9-fold in EGFR mutant patients (*p* < 0.05). The data is sourced from: Hao Liu (2012) [27], Zhenrui Jiang (2018) [28], Guanming Jiang (2019) [29], Yan Li (2016) [30]. (**B**) The distribution of studies in the funnel plot was approximately symmetrical. (**C**) Ginsenoside Rg3 significantly down-regulates EGFR exon 19 in the EGFR-wild-type A549 cell line (*p* < 0.05). (**D**) Ginsenoside Rg3 had no effect on EGFR copy number in the EGFR-wild-type H1299 cell line. (**E**) Ginsenoside Rg3 significantly down-regulated the copy number of exons 18, 19, and 21 of EGFR in H1975 cells (*p* < 0.05). (**F**) Ginsenoside Rg3 significantly down-regulated the copy number of exons 18, 19, and 20 of EGFR in HCC827 cells (*p* < 0.05).

Ginsenoside Rg3 has been widely used in the adjuvant treatment of non-small cell lung cancer [26]. To investigate the increasing role of ginsenoside Rg3 on the efficacy of EGFR-TKI in non-small cell lung cancer, we systematically evaluated the effect of ginsenoside Rg3 in combination with EGFR-TKI in non-small cell lung cancer treatment. The Jadad table was used to evaluate the quality and credibility of the literature. The results are shown in Figure 6A. Ginsenoside Rg3 did not significantly increase the efficacy of EGFR-TKI in the treatment of EGFR-wild-type patients but could increase the objective remission rate of TKI-treated patients up to 1.9-fold in EGFR-mutant patients (OR = 1.47, 95%CI: 1.07–2.23, *p* < 0.05). The distribution of studies in the funnel plot was approximately symmetrical, suggesting no publication bias or other bias in the studies (Figure 6B). This suggests that ginsenoside Rg3 may be more sensitive for patients with the EGFR-mutant phenotype, and EGFR may be an important target for its action. To investigate whether ginsenoside Rg3 exerts this effect through the regulation of EGFR exons, we quantified the copy number changes of 18, 19, 20, and 21 EGFR exons after the action of ginsenoside Rg3. The results showed that ginsenoside Rg3 had a significant down-regulating effect only on the copy number of EGFR exon 19 in the EGFR-wild-type A549 cell line, but not on the copy number of exons 18, 20, and 21 in A549 cells and 18, 19, 20, and 21 EGFR exons in the EGFR-wild-type H1299 cell line (Figure 6C,D). In contrast, for EGFR mutant cell lines, ginsenoside Rg3 significantly down-regulated the copy number of exons 18, 19, 21 of EGFR in H1975 cells and exons 18, 19, 20 of EGFR in HCC827 cells (Figure 6E,F). The present study demonstrates that ginsenosides exert no significant regulatory influence on the copy number of wild-type EGFR; however, they significantly modulate the copy number of mutant EGFR. These findings provide empirical evidence supporting the potential clinical utility of ginsenosides in patients with EGFR mutations and copy number amplifications.

### 2.7. Cell Experiments Again Verified That the Combination of Ginsenosides and Gefitinib Was Superior to Gefitinib Alone

**Figure 7:**
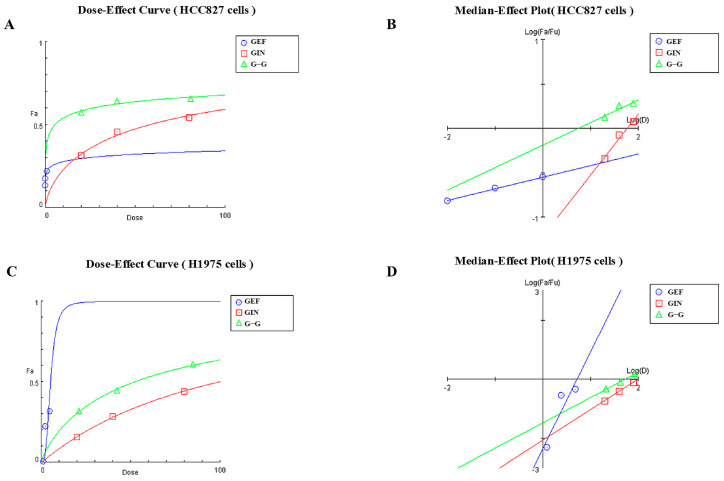
(**A**) Dose–Effect Curve for the HCC827 Cell Line. Blue circles represent the effects of GEF (Gefitinib) alone. Red squares represent the effects of GIN (Ginsenoside Rg3) alone. Green triangles represent the combined effects of GEF and GIN at a 1:1 volume ratio. (**B**) Median–Effect Plot for the HCC827 Cell Line. Blue circles represent the effects of GEF (Gefitinib) alone. Red squares represent the effects of GIN (Ginsenoside Rg3) alone. Green triangles represent the combined effects of GEF and GIN at a 1:1 volume ratio. (**C**) Dose–Effect Curve for the H1975 Cell Line. Blue circles represent the effects of GEF (Gefitinib) alone. Red squares represent the effects of GIN (Ginsenoside Rg3) alone. Green triangles represent the combined effects of GEF and GIN at a 1:1 volume ratio. (**D**) Median–Effect Plot for the H1975 Cell Line. Blue circles represent the effects of GEF (Gefitinib) alone. Red squares represent the effects of GIN (Ginsenoside Rg3) alone. Green triangles represent the combined effects of GEF and GIN at a 1:1 volume ratio.

Based on the results of animal experiments, we designed cellular experiments. Analysis through dose–effect curves and median–effect plots reveals that the combination of GEF (Gefitinib) and GIN (Ginsenoside Rg3), as well as their individual effects, exhibits synergistic inhibitory action on HCC827 and H1975 cell lines. In HCC827 cells, GEF demonstrates dose-dependent inhibitory effects, while GIN shows weaker effects, and the G–G combination exhibits stronger inhibition at low doses (Figure 7A). The median–effect plot indicates synergy, with the G–G combination curve positioned below those of GEF and GIN (Figure 7B). In H1975 cells, GEF has an even stronger inhibitory effect, GIN remains less effective, and the G–G combination again shows superior inhibitory effects at low doses (Figure 7C), with its curve in the median–effect plot also indicating synergy (Figure 7D). Consequently, the G–G combination displays enhanced inhibitory effects in both cell lines, confirming the synergistic interaction between GEF and GIN.

### 2.8. Animal Studies Demonstrate That the Addition of Ginsenosides Significantly Improves the Efficacy of Gefitinib Alone

**Figure 8:**
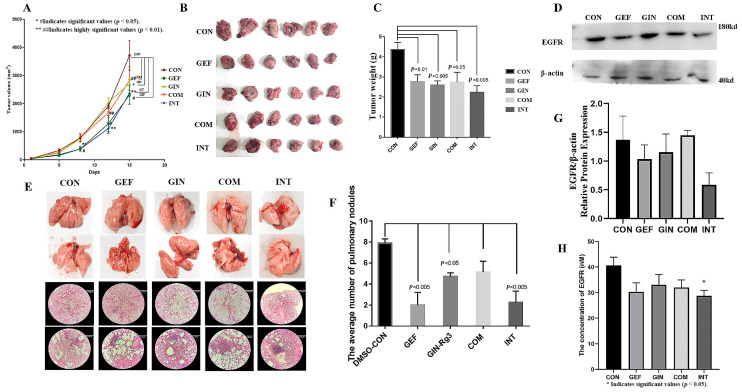
(**A**) The tumor growth curve of the anti-cancer effects of ginsenoside Rg3 or gefitinib alone, the combination regimen, and the gefitinib/ginsenoside Rg3 alternate-day sequential dosing regimen. (**B**) Comparison of tumors in groups of Lewis lung carcinoma-bearing mice. (**C**) The pooled analysis of tumor weight (*p* < 0.05). (**D**) Representative images of EGFR protein expression. (**E**) Pathological sections of lungs from mice with chemically induced lung cancer. Each vertical direction represents the same group. This picture shows example images of the lungs of each group of mice and pathology sections stained with hematoxylin and eosin at 4× and 20× magnification, with the 20× image magnifying the area indicated by the blue arrow in the 4× image. (**F**) The average number of pulmonary nodules (*p* < 0.05). (**G**) Protein expression of EGFR (**H**) The EGFR concentration was measured by ELISA (*p* < 0.05).

Urethane is a commonly used chemical tool for model induction, widely applied in the construction of mouse lung cancer models and capable of causing alterations in exon genes [31]. Whole-exome sequencing reveals Lewis lung carcinoma is a hypermutated Kras/Nras-mutant cancer with extensive regional mutation clusters in its genome [32], therefore, we selected LLC cells for the mouse tumor-bearing experiment. To investigate whether ginsenoside Rg3 could exert potentiating anti-cancer effects on EGFR-TKI agents by down-regulating the copy number and expression of EGFR protein, we performed mouse tumor-loading experiments. Considering that the two drugs may interfere with each other on EGFR as a target, we administered ginsenoside Rg3 and gefitinib [33,34] according to the regimens of continuous dosing alone, combined continuous dosing, and interval sequential dosing of gefitinib/ginsenoside Rg3. The experimental results showed that all four regimens could effectively reduce the tumor growth rate and have significant anticancer effects. The tumor growth curve results also showed that the anti-cancer effects of ginsenoside Rg3 alone and the combination regimen were limited, while the alternate-day sequential dosing regimen of gefitinib and ginsenoside Rg3 can fully achieve the same anti-cancer effects as continuous dosing with gefitinib alone. (Figure 8A). The tumors of the mice in each group were removed and photographed. The tumors in the CON group were significantly larger than those in the other groups (Figure 8B). The pooled analysis of the tumor weights in each group showed that the tumors in each dosing group were significantly smaller than those in the CON group, and the difference was statistically significant (*p* < 0.05) (Figure 8C). The tumors in the INT group of lung cancer-loaded mice were significantly smaller than those in the other dosing groups, which suggests that the continuous dosing regimen has a more significant anticancer effect, with the highest tumor inhibition rate of 48.9%. The expression of EGFR in tumor tissues was examined, and the results of WB experiments also showed that the expression of EGFR in tumor tissues of mice administered with gefitinib alone and gefitinib/ginsenoside Rg3 spaced sequentially was significantly lower relative to the control group. The EGFR content in tumors corresponded to the tumor weight, and the sequential administration group had the lowest EGFR content (Figure 8D). To validate this result, we evaluated the anticancer effects of these regimens again using chemically induced primary lung cancer in mice. Lung tissues from each murine subject were photographed and documented (Figure 8E). Subsequently, a random selection of one specimen per cohort was subjected to hematoxylin and eosin (H&E) staining to elucidate pulmonary nodules and assess the pathological changes. The pulmonary tissues of mice in the control group exhibited severe tumor infiltration, with irregular peripheral spread, substantial alveolar atrophy and collapse, pronounced perivascular fibrous hyperplasia, and significant infiltration of inflammatory cells in the peribronchial and interstitial spaces. Pulmonary tumor invasion was markedly attenuated in mice from other treatment groups, with particularly pronounced inhibition of invasive progression observed in the GEF group and INT group. The enumeration and analysis of lung cancer nodules revealed that sequential administration of ginsenoside Rg3, monotherapy with gefitinib, and intermittent sequential administration of gefitinib combined with ginsenoside Rg3 each significantly decreased the number of lung cancer nodules induced by chemical carcinogens. Specifically, monotherapy with gefitinib and intermittent sequential administration of gefitinib combined with ginsenoside Rg3 reduced tumor incidence by 74.6% and 71.4%, respectively. However, the combination regimen did not exhibit a significant anti-tumorigenic effect. (Figure 8F). Then we confirmed the correlation between the effect of ginsenoside Rg3 and gefitinib when combined with EGFR content, and we examined EGFR protein and EGFR phosphorylation in lung tissues of different groups of mice with chemically induced primary lung cancer. The WB results showed that the expression level of EGFR protein in the lung tissues of mice in the gefitinib/ginsenoside Rg3 interval sequential administration regimen group was the lowest, but there was no significant difference (Figure 8G). The ELISA results showed that both ginsenoside Rg3 and gefitinib tended to reduce EGFR content in lung tissues of mice with carcinoma in situ according to the regimen of continuous administration alone, combined continuous administration, and interval sequential administration of gefitinib/ginsenoside Rg3, respectively, but only the EGFR content in lung tissues of mice with the regimen of interval sequential administration of gefitinib/ginsenoside Rg3 was significantly lower than the control group (Figure 8H, *p* < 0.05). Taken together, the gefitinib/ginsenoside Rg3 interval sequential dosing regimen could fully achieve or be even better than the continuous dosing with gefitinib alone in its anti-cancer effect.

## 3. Discussion

EGFR mutations are one of the most prominent tumor drivers for NSCLC in Asia [35]. Elevated expression of EGFR correlates with tumor cell proliferation, angiogenesis, invasion, metastasis, and the inhibition of apoptosis. Under physiological conditions, the binding of EGFR to extracellular ligands activates downstream signaling pathways, which promote cell differentiation and growth, and are essential for the survival and proper functioning of normal cells. However, the aberrant activation of EGFR can result in the dysregulation of cell proliferation, metastasis, and apoptosis. Specifically, increased EGFR expression may amplify downstream signaling, causing a persistent activation of cell proliferation and survival signals. Exon 18, 19, 20, and 21 mutations are common mutant genotypes in patients with NSCLC, with exon 19 deletions and the L858R point mutation in exon 21 together accounting for approximately 85% of all EGFR mutations in NSCLC. Patients with these mutations showed significant sensitivity to EGFR TKIs, especially first-generation TKIs such as gefitinib, erlotinib, and ectinib [36]. Additionally, the loss, mutation, and rearrangement of EGFR genes can lead to the generation of mutant EGFR variants capable of constitutive receptor activation independent of ligand binding. Upregulation of mutant EGFR receptors or ligands can induce sustained EGFR activation, thereby fostering the proliferation and survival of tumor cells. NSCLC patients with exon 18, 19, 20, and 21 mutations, alterations in EGFR copy number, could potentially disrupt receptor downregulation mechanisms, leading to continuous receptor activation and aberrant signaling pathway activation, which may impact cell cycle regulation and mitotic control. Moreover, EGFR can modulate tumor angiogenesis by influencing the levels of factors such as angiopoietin-1 (Ang-1) and vascular endothelial growth factor (VEGF), consequently affecting the high invasiveness and metastatic potential of tumors [37].

In our study, the correlation between EGFR mutations and NSCLC was investigated in detail, and the significant impact of EGFR structural abnormalities and copy number abnormalities, in addition to gene mutations, on the disease progression and survival prognosis of NSCLC patients was suggested. We found that patients with EGFR abnormalities had a faster disease progression, the probability of tumor cell invasion and metastasis was significantly higher in patients with EGFR abnormalities compared to those with normal EGFR, and there was a high overlap between EGFR mutations and apparent copy number abnormalities, suggesting that DNA copy number abnormalities of EGFR also occur in NSCLC patients with EGFR mutations and have a role in the development of malignant tumors. This suggests that EGFR mutation and DNA copy number abnormalities should be taken into account when targeting EGFR therapy. Several studies have also shown that DNA copy number abnormalities may be an important reason for the development of drug resistance and other problems with EGFR-targeted drugs.

The current clinical treatment for NSCLC with EGFR mutations is strategically more inclined to TKI therapy. However, it is uncertain whether TKI therapy has better results for NSCLC patients compared to the current first-line chemotherapy approaches. In this study, we compared the survival of NSCLC patients who received TKI therapy with NSCLC patients who did not receive TKI therapy. The experimental results showed that although NSCLC patients with abnormal EGFR preferred TKI therapy compared to patients with normal EGFR, clinical EGFR-TKI therapy did not have a significant advantage over first-line chemotherapeutic agents in NSCLC patients with abnormal EGFR based on the available data. Further analysis of EGFR gene amplification copy data in NSCLC patients revealed significant differences in the probability of overall survival between NSCLC patients with different EGFR gene copy numbers, and the higher the increase in EGFR gene copy number, the worse the survival prognosis of the patients. Increasing EGFR copy number is prognostically harmful in NSCLC patients, but EGFR-TKI agents targeting tyrosine receptor activity may not be effective in patients with significantly increased EGFR copy numbers. Because of the high overlap between EGFR mutation and high copy number, the use of EGFR-TKI agents in the treatment of patients with EGFR mutations may need to be complemented by the addition of agents that modulate copy number alterations, taking into account the impact of copy number alterations. Amplicon-based next-generation sequencing data reveals that an increased EGFR copy number prior to treatment is associated with poorer overall survival in patients with EGFR mutations who receive first-line EGFR tyrosine kinase inhibitors [38], and combination therapies targeting EGFR copy number elevations are showing promise, such as EGFR-TKI combined with MET inhibitors [39].

Ginsenoside Rg3 has been widely used in the adjuvant treatment of non-small cell lung cancer [25,40,41]. Several studies have shown that ginsenoside Rg3 shows a significant regulatory effect on EGFR and can reduce EGFR expression and activation in a variety of tumor cells [26]. Our systematic evaluation of the effect of ginsenoside Rg3 in combination with EGFR-TKI in the treatment of non-small cell lung cancer also further validated that ginsenoside Rg3 may be more sensitive for patients with EGFR mutant phenotype and that EGFR may be an important target for its action. In this study, we also found that ginsenoside Rg3 has an effect on down-regulating the copy number of EGFR in both EGFR-wild-type and mutant lung adenocarcinoma cells, but its effect is more pronounced in EGFR-mutant lung adenocarcinoma cells, which may be an important mechanism for its potentiating effect on EGFR-TKI-like agents against cancer. We believe that gefitinib and ginsenoside Rg3 have a common regulatory effect on EGFR, so we designed different combination regimens. Comprehensively, a gefitinib/ginsenoside Rg3 interval sequential dosing regimen was carried out to fully achieve the anti-cancer effect with gefitinib alone continuous dosing, and also further confirmed the correlation between the effect of ginsenoside Rg3 and EGFR content when combined with gefitinib. The combined use of gefitinib and ginsenoside Rg3 has been proven to enhance the inhibitory effects observed when using either drug alone, implying a synergistic effect. In previous clinical studies, it has been confirmed that ginsenoside Rg3 improves median PFS (Progression-Free Survival) and ORR (Objective Response Rate) of first-line EGFR-TKI treatment in patients with EGFR-mutant advanced NSCLC (Non-Small Cell Lung Cancer). This synergy has been consistently observed in both the HCC827 and H1975 cell lines, as evidenced by dose-effect and median-effect plots [42]. Compared to the use of the drugs individually, the G–G combination can achieve a greater inhibitory effect at lower concentrations, which may translate into a more effective treatment strategy and potentially reduce side effects. This result is consistent with clinical studies and further supports that both ginsenoside Rg3 and gefitinib exert their anti-cancer effects through the EGFR pathway. Considering the economic cost and the vulnerability to drug resistance of EGFR-TKI class drugs, this result can provide a reference and basis for optimizing the clinical dosing strategy. Such sequential dosing regimens have been mostly seen in several reports [43,44]. There are many reasons for this phenomenon, and in addition to the competition between drugs acting on the same site, whether the drugs have interactions with each other is also an important consideration. Also, the specific mechanism of the reduction of the important EGFR exon copy number by ginsenoside Rg3 requires further in-depth studies to follow.

There are some shortcomings in this study. Firstly, gefitinib is a first-generation TKI drug used in this study, which is representative, but may have the limitation of weak efficacy and cannot represent the efficacy of the latest generation of TKI in a clinical application. In addition, there is no experimental evidence to prove whether drug toxicity or drug interactions in combination or sequential interval dosing regimens affect efficacy. The survival time and disease progression of patients receiving different treatments were considered in the database mining process, without a comprehensive consideration of patients’ quality of survival and drug dependence that may have arisen.

The irregularities in the EGFR, a key factor in the development of NSCLC, include both genetic mutations within crucial EGFR regions and irregular DNA amplification, which are major contributors to the worsening of the disease and poor patient outcomes. There is a notable correlation between EGFR gene mutations and increased copy numbers, where higher copy numbers can reduce the effectiveness of EGFR-targeted tyrosine kinase inhibitors (TKIs) in patients with the EGFR mutation.

This study also has significant reference value for the treatment with third-generation TKIs. Ginsenosides are gaining attention due to their significant research value and potential applications, particularly in lung cancer treatment, the experimental results of our study provide empirical data support for the clinical combination of TKI and Rg3.

In conclusion, our research shows that ginsenoside Rg3 can reduce the EGFR copy number, especially in the critical exons of EGFR-mutated lung adenocarcinoma cells, and decrease the levels of EGFR proteins, thus enhancing the effectiveness of EGFR-TKIs when used in combination.

## 4. Materials and Methods

### 4.1. Build Database

① Using “lung cancer” as the keyword, we searched the cBioPortal database, selected 31 relevant studies, and obtained 8693 samples from 7994 patients. ② Using non-small cell lung cancer as the screening criteria, we selected “non-small cell lung cancer” and “lung adenocarcinoma” as the screening criteria, and the “custom selection” function was used to retain the selected samples. We obtained 7755 samples from 7155 patients as the non-small cell lung cancer group. ③ The keyword “EGFR” was used to query 8665 samples from 7966 patients in 31 studies, and there were 1930 mutations (including gene mutations, structural variants, and copy amplification) in 1806 samples from 1626 patients, and 629 of the same mutations in multiple patients. The .tsv file was downloaded and imported into Excel. There were 38 samples that did not belong to NSCLC and were therefore deleted, leaving 1892 as the EGFR-abnormal NSCLC group. The IDs of NSCLC patients in the non-small cell lung cancer group and the IDs of NSCLC patients in the EGFR-abnormal gene group were entered into the cBioPortal database in order. Subsequently, “select the currently” option was used to establish the EGFR-abnormal NSCLC group. In the non-small cell lung cancer group, we entered the IDs of NSCLC patients in the EGFR-gene-abnormal group into the cBioPortal database sequentially, and then used “select the currently” to remove EGFR-gene-abnormal samples and create the EGFR-normal NSCLC group.

In the EGFR-abnormal NSCLC group, the samples with an EGFR mutation in exons 18, 19, 20, or 21 were screened to be the EGFR-exon-mutant NSCLC group. The samples without exon mutation were screened as the EGFR-exon-wild NSCLC group. According to their mutation exon, the IDs were set as EGFR-mutation-in-18/28 NSCLC group, EGFR-mutation-in-19/28 NSCLC group, EGFR-mutation-in-20/28 NSCLC group, or EGFR-mutation-in-21/28 NSCLC group. A total of 3734 samples were screened for EGFR-gene-copy-number detection in the EGFR-abnormal NSCLC group data, which were set as EGFR-gain group, EGFR-amplification group, EGFR-shallow-deletion group, EGFR-deep-deletion, and EGFR-diploid group based on their copy number status.

We entered the data for each group in the cBioPortal database separately, and selected samples and cases with EGFR-TKI treatment in the Treatment per patient, where EGFR-TKI drugs included gefitinib\erlotinib\icotinib\afatinib\dacomitinib\osimertinib\almonertinib\furmonertinib\EGF816\rociletinib (CO-1686)\; the data were downloaded as the EGFR-TKI group. Patients/samples using NSCLC first-line chemotherapy agents, including carboplatin\cisplatin\pemetrexed\pabolizumab\paclitaxel\atelizumab\albumin paclitaxel\docetaxel\navumab\imizumab\pamumab\pabolizumab\bevacizumab, were selected in the “Treatment per patient”, and the data were downloaded as the first-line-chemotherapy-drug group.

### 4.2. Meta-Analysis

The CNKI, CSTJ, CDDB, CBM, and PubMed databases were searched from their date of establishment to 1 June 2022. The search terms included “ginsenoside Rg3”, “EGFR”, “gefitinib”, “TKI”, “lung cancer”, “NSCLC”, and “Shenyi Capsules”. The literature inclusion criteria were as follows: ① Study type: domestic and foreign published randomized controlled studies. ② Study subjects: non-small cell lung cancer patients with clinically confirmed EGFR-mutant and wild-type patients; age and gender were not limited. ③ Intervention: the control group was treated with EGFR-TKI, and the experimental group was treated with ginsenoside Rg3 or its medical preparation in combination with EGFR-TKI. Efficacy index: articles with unclear research type; studies with ambiguous endings and indicators for which data could not be extracted; studies of unavailability of full text leading to data extraction; only one of any duplicate published studies was included.

### 4.3. Reagents and Instruments

Ginsenoside Rg3 and gefitinib were both purchased from Aladdin, China; Urethane (Ethyl carbamate) was purchased from Sigma, St. Louis (St. Louis, MO, USA). Rabbit anti-EGFR primary antibodies were purchased from Biosynthesis, China; HRP-labeled and FITC-labeled secondary antibodies were both purchased from Servicebio, China. Forward and inverted integrated fluorescence microscope (Echo Revolve RVL-100) were made by Echo Laboratories, USA (Broomfield, CO, USA).

The cell line was obtained from the Cell Resource Center, Peking Union Medical College (which is part of the National Science and Technology Infrastructure, the National Biomedical Cell-Line Resource, NSTI-BMCR. http://cellresource.cn, accessed on 29 September 2022.). The cell line was tested and authenticated by STR.

### 4.4. CCK8 Assay

CCK8 assay was used to evaluate the half-inhibitory effect of cell lines as described previously [45]. Briefly, cells at a density of 4 × 10^4^ per well were seeded in the 96-well plates for 24 h. Then a gradient of concentration of Rg3 was added. After 72 h, CCK8 was added and incubated for 1 h followed by subjecting the plates using a microplate reader. The combination of drugs was analyzed using CompuSyn 1.0.1, (CompuSyn software, Paramus, NJ, USA), and the combination effect analysis was performed by a dose–effect curve and a median–effect Plot.

### 4.5. Animal Studies

Healthy SPF-grade Balb/c male mice (SPF (Beijing) Biotechnology Co., Ltd., Beijing, China, License No.: SYXK (JING) 2023-0011) were used for the experiments at 6–8 weeks of age, with a body mass of 18–22 g, 12 h/d of light exposure, and unlimited water intake. All animal experiments were approved by the Animal Care and Experimental Committee of Beijing University of Chinese Medicine (No. BUCM-2024062805-2218). The primary tumor model was established using the same method that was reported by Tianhau Liu et al. [46]. A mouse lung cancer model was established by inducing with urethane (0.8 mg/g body weight) dissolved in 0.9% NaCl (saline) twice a week at intervals of 72 h for 5 weeks. For the mouse tumor loading expt, 1 × 10^6^ LLC cells in 100 µL PBS were injected subcutaneously into the right flank of Balb/c mice. Mice were randomly divided into five groups: (1) GIN group received 3 mg/kg ginsenoside Rg3; (2) GEF group received 45 mg/kg gefitinib; (3) CON group received an equal volume of solvent (normal saline with 0.7% DMSO); (4) COM group received 3 mg/kg ginsenoside Rg3 and 45 mg/kg gefitinib; (5) INT group received 3 mg/kg ginsenoside Rg3 or 45 mg/kg gefitinib at sequential intervals. At the end of the experiments, all mice were humanely killed, and lung tissues were photographed and preserved. For the urethane-modeled mice, we counted and enumerated all surface nodules in the upper and lower lobes visible to the naked eye after autopsy (Nodule morphology: white transparent protuberance, hard texture) and reexamined them after fixation in paraformaldehyde. For the Lewis lung carcinoma-bearing mice, during the administration period, tumor volume was measured by a vernier caliper every 2 days. The tumor volume was calculated as follows: tumor volume (mm^3^) = length × width^2^ × 0.5.

### 4.6. Histopathological Analysis

Lung tissues from sacrificed mice of primary tumor model groups were fixed in 4% paraformaldehyde, embedded in paraffin, sectioned at 4–5 µm, and stained with hematoxylin and eosin (H&E) by conventional method and observed under a microscope.

### 4.7. Quantitative Real-Time Polymerase Chain Reactions (RT-qPCR) Assay

The total DNA from cells was extracted according to the kit manual. The reaction conditions were conducted at 94 °C for 1 min; 98 °C for 10 s, followed by 45 cycles at 95 °C for 15 s, 60 °C for 60 s and elongation at 72 °C for 10 min. The primer sequences were as follows: EGFR-18 exon: forward 5′-GAGGTGACCCTTGTCTCTGTGT-3′, reverse 5′-CCCAAACACTCAGTGAAACAAA-3′; EGFR-19 exon: forward 5′-TGCCAGTTAACGTCTTCCTTCT-3′, reverse 5′-TGAACATTTAGGATGTGGAGAT-3′; EGFR-20 exon: forward 5′-ACTTCACAGCCCTGCGTAAAC-3′, reverse 5′-ATGGGACAGGCACTGATTTGT-3′; EGFR-21 exon: forward 5′-GAGCTTCTTCCCATGATGATCT-3′, reverse 5′-GAAAATGCTGGCTGACCTAAAG-3′; GAPDH, forward 5′-ATCAGCAATGCCTCCTGCAC-3′, reverse 5′-CGTCAAAGGTGGAGGAGTGG-3′. GAPDH was used as a reference. The reactions were carried out with SYBR-Green in Step-One Plus RT-qPCR System (Applied Biosystems, Waltham, MA, USA).

### 4.8. Western Blotting

Protein expression was detected using rabbit polyclonal primary antibody of EGFR and appropriate HRP-labeled secondary antibodies. Protein concentration was measured by the standard curve obtained by the Coomassie brilliant blue method. β-actin was used as a loading control. Samples were obtained from three different mice in each group.

### 4.9. ELISA

Lung tissues were from sacrificed mice of primary tumor model groups. After tissue fragmentation, the protein supernatant was extracted and subsequent operations and assays were performed according to the Mouse EGFR ELISA Kit instructions, which were purchased from Wuhan Fine Biotech Co., Ltd. (Wuhan, China).

### 4.10. Statistical Analysis

For the meta-analysis, statistical tests were performed using RevMan 5.0. Relative risk (RR) and 95% confidence intervals (95% CI) were used as statistics for the efficacy analysis, and the I2 test was used to test for heterogeneity. The fixed-effects model was used for meta-analysis when the heterogeneity between studies was small (*p* > 0.10, I2 < 50%). Conversely, when statistical heterogeneity between studies was large (*p* ≤ 0.10, I2 > 50%), subgroup analysis or sensitivity analysis was performed to minimize heterogeneity according to the possible heterogeneity factors. Funnel plots were used to analyze the presence of publication bias. Experiments results were expressed as mean ± standard deviation (SD) and were analyzed by GraphPad Prism 7 (GraphPad Software, San Diego, CA, USA). Statistical analysis was carried out by a two-tailed Student’s *t*-test, one-way ANOVA test or Newman-Keuls test as requested, and *p* < 0.05 was considered significant.

## Data Availability

The data underlying this article are available in cbioportal, at https://www.cbioportal.org; CNKI, at https://www.cnki.net; VIP http://www.cqvip.com/; WANFANG DATA, at http://www.wanfangdata.com/; SinoMed, at http://www.sinomed.ac.cn/; PubMed, at https://pubmed.ncbi.nlm.nih.gov. Accessed on 14 July 2022. The data underlying this article will be shared on reasonable request to the corresponding author.

## Abbreviations

The following abbreviations are used in this manuscript:

EGFR	Epidermal Growth Factor Receptor
TKI	Tyrosine Kinase Inhibitors
NSCLC	Non-Small Cell Lung Cancer
